# Childhood trauma and psychological sub-health among Chinese adolescents: the mediating effect of Internet addiction

**DOI:** 10.1186/s12888-022-04384-2

**Published:** 2022-12-05

**Authors:** Meng Yang, Xuanlian Sheng, Menglin Ge, Ling Zhang, Cui Huang, Shu Cui, Qiuyu Yuan, Mengting Ye, Ruochen Zhou, Panpan Cao, Ran Peng, Xiaoqin Zhou, Kai Zhang

**Affiliations:** 1 School of Mental Health and Psychological Sciences, Anhui Medical University, No. 81 Meishan Road, Hefei, 230032 Anhui China; 2 Department of Psychiatry, Chaohu Hospital, Anhui Medical University, No. 64 Chaohubei Road, Hefei, 238006 Anhui China; 3 Anhui Psychiatric Center, Anhui Medical University, No. 64 Chaohubei Road, Hefei, 238006 Anhui China

**Keywords:** Psychological sub-health, Childhood trauma, Internet addiction, Adolescents

## Abstract

**Background:**

The factors related to psychological sub-health (PSH) have been widely described, but the research on the mechanism behind the complex relationship between childhood trauma and PSH is limited. This study investigated the current situation and risk factors of PSH among Chinese adolescents. And further, explore whether Internet addiction (IA) plays a potential mediating effect in childhood trauma and PSH.

**Methods:**

The study was conducted in October 2020 in Anhui Province, China. The PSH state of 866 adolescents was investigated, including demographic information such as gender, age, and grade. Childhood trauma, IA, and PSH were measured by the Childhood Trauma Questionnaire Short Form (CTQ-SF), Young’s Internet Addiction Test (IAT), and the Multidimensional Sub-health Questionnaire of Adolescents (MSQA). The mediating effect is further verified by the structural equation model (SEM).

**Results:**

In this study, 866 adolescents were selected as subjects, and the proportion of male and female is roughly equal. The prevalence of PSH in adolescents was 25.8%, and left-behind children, boarding, or adolescents who have had non-suicidal self-injury (NSSI) are more likely to have PSH. Through the mediation test, the direct effect of childhood trauma on PSH was 0.23 (95% CI [4.91,9.00],*p <*0.001), and the mediating effect of IA on childhood trauma and PSH was 0.07 (95% CI [1.42, 3.32],*p <*0.001). and the proportion of them is 75.14% and 24.86% respectively.

**Conclusions:**

Childhood trauma has direct and indirect effects on PSH, and IA plays a mediating effect in the indirect effect. Therefore, clarifying these relationships helps formulate and implement effective interventions to improve psychological health (PH) in Chinese adolescents.

## Introduction

Psychological sub-health (PSH) refers to the transition state between illness and health. The main manifestations are unexplained mental fatigue, mood disorders, confusion, panic, anxiety, neuroticism, low self-esteem, apathy, loneliness, recklessness, and even suicidal thoughts [1]. Since the psychological state of adolescents is in the stage of transition from immaturity to maturity, their psychological health (PH) is easily affected by the external environment. As estimated, 10% to 20% of children and adolescents worldwide suffer from PH problems [2]. The prevalence rate of PSH state among adolescents in China is 15.50% [3].

This has had a serious impact on the daily lives and learning of children and adolescents during the COVID-19 pandemic. They may face more family conflict, parent-child conflict, and abuse; Loss of prosocial activities; Adapt to online education; Increased screen time, increased sedentary time, etc. The results showed an increase in anxiety and depressive symptoms among adolescents compared to pre-pandemic levels [4, 5]. During this period, the mental health of more than 20% of Chinese adolescents was affected [6]. Behavioral and emotional problems appear to be more prevalent than in the past [7, 8].

Childhood trauma is defined as adverse life events experienced between birth and 18 years of age that [9], if poorly managed, can become risk factors for PSH, such as anxiety, depression, and self-harm/suicide [10–13]. Its incidence is high, with up to two-thirds of the population experiencing adverse childhood trauma before the age of 18 [14]. The prevalence of adverse childhood trauma is higher in China, with the prevalence of adolescents reaching 79.01% [15]. Childhood trauma is a common risk factor for lifelong psychosomatic diseases. Different mediating mechanisms contribute to these negative effects, including biological regulatory mechanisms and psychosocial mechanisms [16]. Childhood trauma crushes the victim, undermining their inner schema of security and trust and becoming more sensitive, thereby undermining their ability to concentrate, in the long run, hindering their PH [17]. Comprehensive studies have found that adolescents with childhood trauma have certain problems in PH dimensions, such as emotional regulation disorder, interpersonal instability, poor coping ability, and cognitive dysfunction [18, 19]. Studies have found that childhood trauma can lead to a variety of emotional and behavioral problems, including depression, substance abuse, increased risk-taking behavior, increased aggression, relationship difficulties, the development of post-traumatic stress disorder (PTSD), and self-harm and suicidal thoughts [20, 21]. Childhood trauma has been linked to lower PH levers and more severe PH problems, and the higher the frequency of adverse childhood trauma, the higher the incidence of PH problems [22, 23].

Adolescents with childhood trauma who grow up in an unsafe home environment may self-medicate with negative emotions through excessive Internet use, eventually leading to Internet addiction (IA) [24]. In neurophysiology, since the family stressor (childhood trauma) is negative stress, it will disrupt the stress response system and change the neural structure and function over time, thus increasing the susceptibility to IA [25, 26]. IA is one’s ability to use the Internet that cannot be controlled [27, 28]. This use of the Internet is excessive and morbid [29, 30]. In addition, IA is negatively correlated with PH in adolescents, such as emotional and conduct problems, which are also two dimensions for evaluating PSH. And it can even lead to more serious mental disorders such as anxiety, depression, and hyperactivity [31–34]. Overuse of the Internet is a strategy used by adolescents who have experienced childhood trauma to cope with stress, and this strategy reinforces adolescent overuse of the Internet, which can easily lead to IA among adolescents [24]. At the same time, excessive use of the Internet can interfere with teenagers’ physical and mental health, leading to psychological problems [35, 36]. Based on the above, we hypothesize that IA may be an underlying risk for the development of PSH from childhood trauma. That is, IA plays a mediating effect between childhood trauma and PSH.

In conclusion, PSH has a high incidence and great harm. Childhood trauma, IA, and PSH are closely related, the individual relationships between childhood trauma, IA, and PSH have been reported in previous studies. However, few studies have explored the role of IA in the relationships between childhood trauma and PSH. Therefore, the primary purpose of this study was to describe the overall PSH status of adolescents in Anhui Province. The second purpose of this study was to investigate the specific relationship between childhood trauma, IA, and PSH. Finally, to explore whether IA plays a mediating role in the relationship between childhood trauma and PSH. The study was designed to raise public awareness, to provide theoretical support for the prevention of PSH in adolescents.

## Materials and methods

### Participants

This survey was approved by the Ethics Committee of Chaohu Hospital of Anhui Medical University(2019-kyxm-012). First of all, two cities were randomly selected in Anhui province, and then students from six schools in these two cities were randomly selected as the research objects, from October 2020 to October 2021. Participants in this survey should meet the following conditions: (1) minors (< 18 years old), and (2) volunteer to participate in this survey. Exclusion criteria: adolescents with severe mental or physical illness or impaired audiovisual function. The survey required informed consent from participants and their guardians (parents or other caregivers) before the survey. Then, before filling out the relevant scale, the researcher explained the process and purpose of this study and gave unified instructions to the participants, and then the participants filled out the questionnaire anonymously and independently.

### Measures

#### General sociodemographic data

The participants’ age, gender, grade, the status of left-behind children, whether they are only children, parent’s education level, parent’s marital status, family economic conditions, and so on were investigated.

#### Multidimensional sub-health questionnaire of adolescent (MSQA)

The PSH status was evaluated by the MSQA, which included 39 items, including three dimensions: emotional problems, conduct problems, and social adaptation difficulties. Items with sub-health symptoms lasting more than 1 month (i.e., score > 4) are considered positive items. The higher the score, the longer duration of sub-health symptoms, and the number of positive items ≥8. Then, the participant is assessed to be in the PSH state [37]. The scale has good reliability and validity and is widely used in China [38, 39]. The Cronbach’s α coefficient value was 0.90 in this study.

#### Childhood trauma questionnaire short form (CTQ-SF)

The simplified version of the CTQ-SF includes 28 items, the symptoms ranged from never (1) to always (5). The higher the score, the more traumatic experience, and the more abuse. The scores of moderate and severe in each subscale were as follows: emotional abuse > 12, physical abuse > 9, sexual abuse > 7, emotional neglect > 14, physical neglect > 9. Participants with a moderate or severe self-assessment score were marked 1 on each of the five subscales, indicating that they had suffered this type of trauma [40]. CTQ-SF has been widely used in Chinese adolescents and has been proven to have good reliability and validity [38, 41]. The scale was found to be reliable in this study (Cronbach’s alpha = 0.73).

#### Young’s Internet addiction test (IAT)

The IAT consists of 20 items designed to measure IA. These items were rated on a scale of 5, from 1(rarely) to 5(always). The total score is 20 to 100 points. The higher the score, the deeper the IA. According to the criteria, 20-49 points are normal, and ≥ 50 points are mild to severe IA [42, 43]. Its Chinese version has good reliability and validity and Cronbach’s α coefficient was 0.90 [44, 45].

### Statistical analysis

SPSS 25.0 software was used for statistical data analysis in this study. According to the calculation method of the scale, MSQA was transformed into a dichotomous variable and divided into the PSH group and the PH group. All data were tested for normality by the Shapiro-Wilk test and did not follow the normal distribution. Mann-Whitney U test (continuous variables) and Chi-square test (categorical variables) were used to analyze general sociological characteristics between the two groups. According to the scale calculation method, the CTQ-SF and IAT were transformed into dichotomous variables, and the chi-square test was used to test the differences between the PSH group and the PH group. The Mann-Whitney U test was used to compare the subscale of CTQ-SF (continuous variables) and the total score of IAT between the two groups. Spearman correlation was used to analyze the correlation between the three variables. Binary logistic regression was used to analyze the influence of general sociological characteristics, childhood trauma, and IA on PSH.

The structural equation model (SEM) was constructed using AMOS 24.0 software to test the hypothesis that IA plays a mediating role in the relationship between childhood trauma and PSH among adolescents. PSH is a latent variable, which consists of emotional problems, behavioral problems, and social adaptation difficulties. Specifically, the bias-corrected 95% confidence interval (CI) was calculated using the bootstrap method for 2000, and indirect effects were considered significant if the bootstrap 95% CI did not include 0. The degree of fitting of the hypothesis model to sample data is evaluated by the following goodness-deviation measures: Chi-square degrees of freedom ratio (CMIN/DF), Goodness-of-fit index (GFI), Adjusted goodness-of-fit index (AGFI), Normal fit index (NFI), Relative fit index (RFI), Incremental fitting index (IFI), Tacker-Lewis’s index (TLI), Comparative fitting index (CFI) and Root mean square error of approximation (RMSEA). If the CMIN / DF value is less than 2, the values of GFI, AGFI, NFI, RFI, IFI, TLI, and CFI, are above 0.9, and the RMSEA value is less than 0.08, the model fits well. All the Significance was set at 2-tailed*P*< 0.05.

## Results

### Socio-demographic characteristics of participants

A total of 937 questionnaires were collected, and 866 were valid, with an effective response rate of 92.4%. As shown in Table 1, the number of participants with PSH was 223, and the prevalence was 25.8%. The proportion of males and females was about the same, with an average age of 12.96 ± 1.50 years (range, 10-17 years). Most of the participants were middle school students, a total of 516. Most of the participants (59.0%) had siblings, that is, non-only children. The parents’ education level is mostly junior high school or below, and the family economic condition is general. Unfortunately, 16.7% of their parents were divorced, or one of their parents died. According to statistics, about half of the participants are left-behind children (48.8%) and 30.9% of participants are residential students. There were statistically significant differences between the PSH group and PH group in whether they were left-behind children and accommodation type (*p *< 0.001). Among all the participants, 308 (35.6%) had non-suicidal self-injury (NSSI), The detection rate of non-suicidal NSSI in the PSH group was significantly higher than that in the PH group (65.5% vs 25.2%, *p*< 0.001). It can be seen that the PH group and the PSH group have no statistical significance in age, gender, grade, and only child, and parents’ marital status, education level, and family economic status were also not statistically significant.

**Table 1:** Socio-demographic characteristics of adolescents

Variables	Total Participants (*n*= 866)	PSH (*n*= 223,25.8%)	PH (*n*= 643,74.2%)	Z/X^2^	*P*
Age	12.96 (1.50)	13.05 (1. 52)	12.92 (1.50)	1.15	0.25
Gender				3.23	0.07
Male	441 (50.9%)	102 (45.7%)	339 (52.7%)		
Female	425 (49.1%)	121 (54.3%)	304 (47.3%)		
Grade				2.57	0.11
Primary school	350 (40.4%)	80 (35.9%)	270 (42.0%)		
Middle school	516 (59.6%)	143 (64.1%)	373 (58.0%)		
Left behind status				17.72	**<0.001**
Yes	423 (48.8%)	136 (61.0%)	287 (44.6%)		
No	443 (51.2%)	87 (39.0%)	356 (55.4%)		
Only child				0.01	0.93
Yes	355 (41.0%)	92 (41.3%)	263 (40.9%)		
No	511 (59.0%)	131 (58.7%)	380 (59.1%)		
Father’s educational level				2.96	0.09
<9 years	525 (60.6%)	146 (65.5%)	379 (58.9%)		
≥9yeaes	341 (39.4%)	77 (34.5%)	264 (41.1%)		
Mother’s educational level				1.22	0.27
<9 years	560 (64.7%)	151 (67.7%)	409 (63.6%)		
≥9 years	306 (35.3%)	72 (32.3%)	234 (36.4%)		
Parents’ marital status				3.25	0.07
Married	721 (83.3%)	177 (79.4%)	544 (84.6%)		
Parental divorce or death of a parent	145 (16.7%)	46 (20.6%)	99 (15.4%)		
Economic situation				3.33	0.19
Better	192 (22.2%)	47 (21.1%)	145 (22.6%)		
General	625 (72.2%	158 (70.9%)	467 (72.6%)		
Poor	49 (5.7%)	18 (8.1%)	31 (4.8%)		
Accommodation type				13.00	**<0.001**
Yes	193 (22.3%)	69 (30.9%)	124 (19.3%)		
No	673 (77.7%)	154 (69.1%)	519 (80.7%)		
NSSI				117.21	**<0. 001**
Yes	308 (35.6%)	146 (65.5%)	162 (25.2%)		
No	558 (64.4%)	77 (34.5%)	481 (74.8%)		

*PSH: *psychological sub-health, *PH: *psychological health, *NSSI: *Non-suicidal self-injury. The standard deviation and the percentage are in parentheses. Bolded *P *values < 0.05

### Statistical description and comparison of childhood trauma and IA between PSH group and PH group

As can be seen from Table 2 that more than half of the adolescents (57.5%) have had childhood trauma. The detection rate of childhood trauma was significantly higher in the PSH group than in the PH group (76.2% vs 51.0%, *p*<0.001). Child trauma total score, physical neglect score, and emotional neglect score, such as the five factors in adolescents with PSH, were also significantly higher than those in the PH group (*p*< 0.001). In addition, among 866 subjects, the overall prevalence of mild to severe IA was 23.2%, and the prevalence of IA was significantly higher in the PSH group than in the PH group, and there was a statistical difference between the two groups (52.0% vs 13.2%, *p*< 0.001). The total score of IA in the PSH group was significantly higher than that in the PH group (51.18 ± 17.50 vs 35.04 ± 12.04, *p*< 0.001).

**Table 2:** Prevalence of childhood trauma and Internet addiction and comparison between groups

Variables	Total Participants (*n*= 866)	PSH (*n*= 223)	PH (*n*= 643)	Z/X^2^	*P*
Childhood trauma	50.29 (14.25)	58.20 (15.54)	47.5 (12.68)	−9.48	**<0.001**
Yes	498 (57.5%)	170 (76.2%)	328 (51.0%)	43.12	**<0.001**
No	368 (42.5%)	53 (23.8%)	315 (49.0%)		
Physical neglect	9.57 (3.26)	10.86 (3.37)	9.12 (3.10)	−6.55	**<0.001**
Emotional neglect	11.98 (5.05)	13.45 (5.46)	11.46 (4.79)	−4.87	**<0.001**
Physical abuse	6.50 (3.06)	7.58 (4.04)	6.12 (2.53)	−6.28	**<0.001**
Emotional abuse	7.66 (3.50)	9.85 (4.43)	6.90 (2.73)	−10.55	**<0.001**
Sexual abuse	5.93 (2.61)	6.51 (3.41)	5.73 (2.23)	−4.49	**<0.001**
Internet addiction	39.19 (15.37)	51.18 (17.50)	35.04 (12.04)	−12.43	**<0.001**
Yes	201 (23.2%)	116 (52.0%)	85 (13.2%)	139.85	**<0.001**
No	665 (76.8%)	107 (48.0%)	558 (86.8%)		

*PSH: *psychological sub-health, *PH: *psychological health. The standard deviation and the percentage are in parentheses. Bolded *P *values < 0.05

### The relationships among childhood trauma, PSH state, and IA

Spearman correlation analysis results of childhood trauma, PSH, and IA scores are shown in Table 3. The total score of childhood trauma was positively correlated with the scores of emotional problems (r = 0.41, *p <*0.01), behavioral problems (r = 0.40, *p <*0.01), and social adaptation difficulties (r = 0.40, *p <*0.01). There was also a positive correlation between childhood trauma and IA (r = 0.36, *p *< 0.01). In addition, the relationship between IA and emotional problems (r = 0.50, *p *< 0.01), behavioral problems (r = 0.52, *p *< 0.01), and social adaptation difficulties (r = 0.56, *p *< 0.01), was also a significant positive correlation. There was also a correlation between the subscales of childhood trauma and PSH (min r = 0.19, max r = 0.83, all *p *< 0.01). Therefore, the mediating effect can be further analyzed.

**Table 3:** Relationships among childhood trauma, Internet addiction, and psychological sub-health state. (n = 866)

Variables	1	2	3	4	5	6	7	8	9	10
1. Childhood trauma	1.00									
2. Physical neglect	0.71^**^	1.00								
3. Emotional neglect	0.83^**^	0.49^**^	1.00							
4. Physical abuse	0.55^**^	0.26^**^	0.32^**^	1.00						
5. Emotional abuse	0.65^**^	0.32^**^	0.38^**^	0.51^**^	1.00					
6. Sexual abuse	0.45^**^	0.25^**^	0.24^**^	0.44^**^	0.36^**^	1.00				
PSH state										
7. Emotional problems	0.41^**^	0.29^**^	0.23^**^	0.24^**^	0.47^**^	0.19^**^	1.00			
8. Behavioral problems	0.40^**^	0.30^**^	0.22^**^	0.25^**^	0.49^**^	0.21^**^	0.83^**^	1.00		
9. Social adaptation problems	0.40^**^	0.30^**^	0.22^**^	0.27^**^	0.45^**^	0.22^**^	0.82^**^	0.77^**^	1.00	
10. Internet addiction	0.36^**^	0.27^**^	0.21^**^	0.22^**^	0.38^**^	0.22^**^	0.50^**^	0.52^**^	0.56^**^	1.00

PSH: psychological sub-health **p< 0.01

### Risk factors of PSH state

Risk factors for adolescent PSH state were analyzed by binary logistic regression (Table 4), NSSI (OR = 4.03, CI = 2.82 ~ 5.76, *p <*0. 001), childhood trauma (OR = 1.95,95% CI = 1.32 ~ 2.89, *p *= 0. 001) and IA (OR = 5.20, CI = 3.55 ~ 7.60, *p *< 0. 001) are the risk factors of PSH. Specifically, the PSH of adolescents with NSSI was 4.03 times higher than that of adolescents without NSSI, and participants with childhood trauma were 1.95 times higher than those without childhood trauma. The incidence of PSH with IA is 5.20 times higher than that of adolescents without IA.

**Table 4:** Risk factors for psychological sub-health (*n*= 866)

	B	SE	Wals	*P*	OR	95%CI	
						Lower	Upper
Left behind	0.31	0.19	2.69	0.10	1.36	0.94	1.97
Accommodation	0.03	0.22	0.02	0.89	1.03	0.68	1.57
NSSI	1.39	0.18	58.30	**<0.001**	4.03	2.82	5.76
Childhood trauma	0.67	0.20	11.26	**0.001**	1.95	1.32	2.89
Internet addiction	1.65	0.19	71.86	**<0.001**	5.20	3.55	7.60
Constant	−2.76	0.21	179.17	**<0.001**	0.06		

*SE*standard error,*OR*odds ratio,*CI*confidence interval,*NSSI*Non-suicidal self-injury. Bolded*P*values < 0.05

### The mediating effect of Internet addiction

**Fig. 1:**
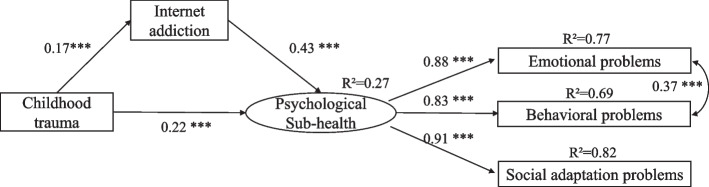
Structural equation model (standardization)

According to the hypothesis of this study, the latent variable SEM is constructed using AMOS24.0 (Fig. 1). The independent, mediating, and dependent variables of the structural equation are childhood trauma, IA, and PSH in this SEM. Since the fitting degree of the directly used model was not good enough, the one-shot MI modification method was used in this study. The modified results show that the fitting condition of the model is good (CMIIN/DF = 1.139, GFI = 0.998, TLI = 0.999, CFI = 1.00, RMSEA = 0.013) and is acceptable.

As in Table 5, the model results showed that the path from childhood trauma to PSH (β = 0.22, t = 6.96, *p <*0.001; 95%CI [0.17,0.30]); From Childhood trauma to IA (β = 0.17, t = 5.19, *p<*0.001; 95%CI [0.11**,**0.23]); From IA to the PSH state of adolescents (β = 0.43, t = 12.74, *p <*0.001; 95%CI [0.36,0.50])**, **these three paths were significant at*P*< 0.001 level.

**Table 5:** The path inspection

Path inspection	Non-standardized coefficient	Standardization coefficient	S.E.	C.R.	*P*
Childhood trauma→IA	0.15	0.17	0.03	5.19	***
IA → PSH	15.36	0.43	1.21	12.74	***
Childhood trauma→PSH	6.89	0.22	0.99	6.96	***

*SE*standard error.*C. R*Divide the regression weight estimate by the estimate of its standard error.*IA*Internet addiction.*PSH*Psychological Sub-Health. ***p< 0.001

In this study, the Bootstrap method (sampling 2000 times at the 95% confidence interval) was used to test the mediating role of IA. The results are as follows (Table 6): The mediating effect of IA on childhood trauma and PSH was 0.07 (SE = 0.47, 95% CI [1.42, 3.32],*p <*0.001). and the proportion 24.86%. Thus, the mediating effect of IA on childhood trauma and PSH is significant.

**Table 6:** Analysis of mediating of Internet addiction, direct effect, and total effect

	Effect value (Standardization)	SE	Bias-corrected 95%CI		*P*	Ratio of effect
			Lower	Upper		
Mediating effect of IA	0.07	0.47	1.42	3.32	**0.001**	24.86%
Direct effect	0.23	1.03	4.91	9.00	**0.001**	75.14%
Total effect	0.30	1.10	6.99	11.28	**0.001**	

*SE* standard error. Independent variables, dependent variables, and mediating variables are childhood trauma, psychological sub-health, and Internet addiction, respectively.*IA*Internet addiction. Bolded*P*values < 0.05

## Discussion

There was a Chinese study that suggested that the COVID-19 pandemic has a significant impact on the PH of children and adolescents [46]. This study, which is still in the COVID-19 phase, showed the PSH prevalence of 25.8%, significantly higher than previously predicted. Our survey results show that left-behind children, boarding students, and adolescents with NSSI are more likely to be in the PSH state. Further studies found that childhood trauma and IA increased the risk of PSH by 1.95 and 5.20 times. And SEM also verified the initial hypothesis that childhood trauma has a positive predictive effect on PSH directly or indirectly through IA. In other words, IA plays a partial mediating effect in the relationship between childhood trauma and PSH.

Left-behind children refer to children aged 0 to 18 who cannot live with their parents because one or both parents have moved to other places for various reasons [47]. Compared with ordinary children, left-behind children are more introverted, withdrawn, and quiet, do not readily show their inner thoughts and feelings, are more apathetic and depressed, are indifferent to things, and have less interested and concerned about things [48]. These children are prone to a series of psychological and behavioral problems [49–51]. Our study also found that left-behind children were more likely to be in the state of PSH than children with parents around. A previous study in China showed a higher prevalence of psychological problems among junior high school students in boarding schools than in non-boarding schools [52]. A survey of American Indian adolescent boarders showed that boarders had significantly higher suicide rates than non-boarders [53]. According to the investigation, boarding schools do not pay enough attention to PH. The reason may be that the complete PH education system has not been formed, the allocation of teachers for PH education is not enough, and the development of PH education is not perfect, so it is necessary to strengthen PH education in boarding schools.

NSSI refers to the intentional self-destruction of body tissue, which is not approved by society. Including cutting, burning, biting, and scratching the skin, without suicidal intention [54]. The incidence of NSSI among adolescents is about 17.2% globally [55], and 23.2% in China [56]. The prevalence of NSSI was 35.6% in this study, which was significantly higher than in previous studies. Some researchers have demonstrated the relationship between NSSI and PSH. This study also inversely verified that NSSI behavior is a predictor and risk factor of PSH in adolescents. Parents or schools are called on to speculate about more insidious PH problems in adolescents through easily detected abnormal behaviors and provide psychological help to avoid worse things happening.

We investigated the participants about childhood trauma, which refers to a chronic form of parental abuse and, or neglect, that is distinct from acute childhood trauma (e.g., traumatic accidents, single assaults, etc.) associated with PTSD [57]. Numerous studies have linked emotional, physical, and sexual abuse to higher rates of PH problems. The results of this study showed that the majority (57.5%) of participants had at least one type of childhood trauma, which significantly increases the risk of PSH in adolescents. This is consistent with previous studies, several studies in China have confirmed that childhood trauma is an independent risk factor for PSH [58, 59]. Many previous studies have suggested that gene expression, abnormal epigenetic changes, abnormal brain structure and function, and hypothalamic-pituitary-adrenal (HPA) axis dysfunction may be the essential biological basis for the relationship between childhood trauma and physical and PH problems in later life [60, 61]. Recently, the study of inflammatory factors has also received extensive attention. Related studies have found that abnormal levels of inflammatory cytokines (TNF-α, IL-6, and acute C-reactive protein) and telomere length may be important biological indicators of physical and psychological health problems in individuals with childhood trauma [62–65]. In the long term, childhood trauma not only increases the incidence of PSH among adolescents, but more seriously, a lot of (but not all) childhood trauma is also associated with multiple types of mental disorders in the course of life, mainly primary mental disorders. They will continue into adulthood [66].

In this study, IA was used as an innovative point to explore the relationship between childhood trauma and adolescents’ PSH state. IA is the excessive and morbid use of the Internet, which not only leads to lower grades, but also increases levels of depression, anxiety, and reduced PH [31, 67]. Especially, with the emergence and increase of online courses, adolescents have more opportunities to access the Internet and spend more time online, which may also be one of the reasons for the increase in the prevalence of IA among adolescents [68–70]. This is also a concern for many parents. In our clinical work, we find that many parents come to consult because their children are addicted to the Internet. In this study, we surveyed participants for IA and found that one in five participants had IA, which greatly increased the risk of PSH. In addition, SEM confirms that childhood trauma has a direct effect on PSH and can also lead to PSH indirectly through IA. As a result of IA, time spent on other personal and social activities decreases, including time spent with friends or family. The problem may develop into more PH problems such as loneliness and depression [71, 72]. However, many longitudinal studies have shown a bidirectional relationship between IA and PH in adolescents, where both can be both cause and effect. It’s a vicious circle. This study was cross-sectional, and it is difficult to determine causality, so the longitudinal survey calculating the incidence rate has more excellent academic value [73]. Many studies have confirmed that childhood trauma is associated with substance addiction and gambling [74]. However, there are few studies on its relationship with IA. In a Turkish study on the risk and severity of IA in college students, emotional abuse appeared to be a major predictor of increased risk of IA among childhood trauma types [33]. Physical abuse has been considered as a possible risk factor for IA in Chinese students [75]. The above reminds us that it is also important to assess the type of childhood trauma in adolescents when considering IA. For example, a cross-sectional survey of Chinese adolescents and young adults found that sexual and emotional abuse had the greatest impact on IA among women. This has practical implications for administrators and educators, reminding them to pay close attention to women who have experienced childhood trauma to reduce the risk of IA [24].

The results of the current study are also limited by methodological factors. First, the study used a cross-sectional design, so the causality is uncertain. To determine the causality, it would be of interest to conduct a prospective study of the sequential assessment of IA and PSH. Secondly, our study sample included only adolescents from two cities in Anhui province, so the results may not be well generalizable to the whole of China or other countries. Thirdly, although the sample size was 866, it was not large enough. Finally, given that all variables were assessed subjectively, participants may have exaggerated or weakened impressions and assessments of childhood trauma.

## Conclusion

In conclusion, PSH is related to childhood trauma and IA, and IA plays a mediating effect between childhood trauma and PSH. For adolescents who have experienced childhood trauma, educational institutions and health services should recognize childhood trauma early and provide relevant education and interventions to prevent further development, thereby improving PH. In addition, clinicians should be reminded to consider childhood trauma and IA problems in the face of adolescent PH (e.g., anxiety, depression), which can help provide effective interventions [76–78].

## Data Availability

As this study is still ongoing, the raw datasets for the current study will not be available until the end of this research project. The data used for this study are available from the corresponding author upon reasonable request.

## Abbreviations

PSH: Psychological sub-health
IA: Internet addiction
CTQ-SF: Childhood Trauma Questionnaire Short Form
IAT: Young’s Internet Addiction Test
MSQA: Multidimensional Sub-health Questionnaire of Adolescent
SEM: Structural equation model
NSSI: Non-suicidal self-injury
PH: Psychological health
PTSD: Post-traumatic stress disorder
